# Interferon β protects against avascular osteonecrosis through interleukin 6 inhibition and silent information regulator transcript-1 upregulation

**DOI:** 10.18632/oncotarget.23337

**Published:** 2017-12-16

**Authors:** Kyoung Min Kim, Sajeev Wagle, Young Jae Moon, Sung Il Wang, Byung-Hyun Park, Kyu Yun Jang, Jung Ryul Kim

**Affiliations:** ^1^ Department of Pathology, Chonbuk National University Medical School, Research Insitute for Endocrine Sciences and Research Insitute of Clinical Medicine of Chonbuk National University–Biomedical Research Insitute of Chonbuk National University Hospital, Jeonju 54896, Republic of Korea; ^2^ Department of Orthopaedic Surgery, Chonbuk National University Medical School, Research Insitute for Endocrine Sciences and Research Insitute of Clinical Medicine of Chonbuk National University–Biomedical Research Institute of Chonbuk National University Hospital, Jeonju 54896, Republic of Korea; ^3^ Department of Biochemistry, Chonbuk National University Medical School, Research Institute for Endocrine Sciences, Jeonju 54896, Republic of Korea

**Keywords:** ischemic osteonecrosis, interferon-β, silent information regulator transcript-1, interleukin 6, osteoclast

## Abstract

Synovitis of the affected joint is a common in avascular osteonecrosis (AVN). Increased levels of pro-inflammatory cytokine interleukin-6 (IL-6) have been reported in AVN, but the mechanism of this increase remains unclear. Silent information regulator transcript-1 (SIRT1), an NAD-dependent deacetylase, inhibits the release of inflammatory cytokines. Interferon β (IFN-β) has clear anti-inflammatory properties. We sought to investigate the effects of IFN-β treatment on AVN and to evaluate the specific signal pathway relating to IL-6 and SIRT1 affected during AVN. Using a dissection microscope, AVN was surgically induced in the distal femurs of mice. Exogenous IFN-β was administered to the model mice. The effects of exogenous IFN-β on AVN model mice were assessed using hematoxylin eosin and safranin-O staining, and bone resorption activity was measured using tartrate-resistant acid phosphatase (TRAP) and CD68 staining. Western blots, real-time RT-PCR, and immunohistochemical staining were performed to evaluate the production of SIRT1 and IL-6 in tissues. The RAW 264.7 cell line and bone marrow derived osteoclasts treated with exogenous IFN-β. Histological findings indicated well preserved trabecular bone and decreased osteoclast bone resorption activity in IFN-β treated mice compared with mice in the AVN group. Treatment with IFN-β increased SIRT1 expression and inhibited secretion of IL-6 in this AVN mouse model. IFN-β decreased IL-6 secretion by activating SIRT1 in the RAW 264.7 cell and bone marrow derived osteoclasts. Our work suggests that IFN-β could be used to treat AVN and that both SIRT1 and IL-6 are useful targets for treating patients with AVN.

## INTRODUCTION

AVN, also known as osteonecrosis, or ischemic bone necrosis is a condition that causes bone destruction progressively as a result of compromised blood vessels of the bone, death of bone tissue and bone marrow cells [1–3]. Although a number of risk factors are associated with the disease, its etiology remains unknown [4–8].

Synovial inflammation in patients with AVN is common, although the mechanism of the inflammation remains unclear [9]. According to a previous study, AVN patients have inflamed synovial tissues without having an inflammatory disease [10]. Recent genetic study found significantly overrepresented heterozygous subjects with an IL-6 polymorphism in a control group compared to the Legg-Calvé-Perthes disease (childhood form of ischemic osteonecrosis) group [11]. Another study reported that the protein level of the major proinflammatory cytokine IL-6 in synovial fluid was significantly elevated in Legg-Calvé-Perthes disease [12]. Therefore, synovial inflammation may play a role in disease progression in the pathogenesis of AVN. Meanwhile, SIRT1, NAD-dependent histone deacetylase, regulates a variety of physiological processes via the cell cycle, metabolism, and inflammation [13–16]. SIRT1 inhibits NF-kB transactivational activity by deacetylation. SIRT1 deacetylates Lys310 of the RelA/p65 subunits of NF-kB and inhibits both its transcriptional activity and the release of inflammatory cytokines mediated by NF-kB [16]. Therefore, SIRT1 may regulate the inflammatory response.

IFN-β has clear anti-inflammatory properties; moreover, it plays an important role in maintaining bone homeostasis by inhibiting osteoclastogenesis. After IFN-β administration in the collagen induced arthritis model, synovial inflammation, cartilage, and bone destruction were clearly attenuated [17]. We hypothesized that exogenous IFN-β would prevent bone resorption and preserve the structural integrity in AVN and that the anti-inflammatory properties of the SIRT1 could represent a link between ischemic necrosis of the bone and bone remodeling. The purposes of this study were to determine whether exogenous IFN-β treatment could inhibit bone resorption and preserve bone morphology in a murine model of AVN and to evaluate the signal pathway affected during AVN with regard to IL-6 and SIRT1.

## RESULTS

### Micro-CT assessment and bone histomorphometric analysis

**Figure 1:**
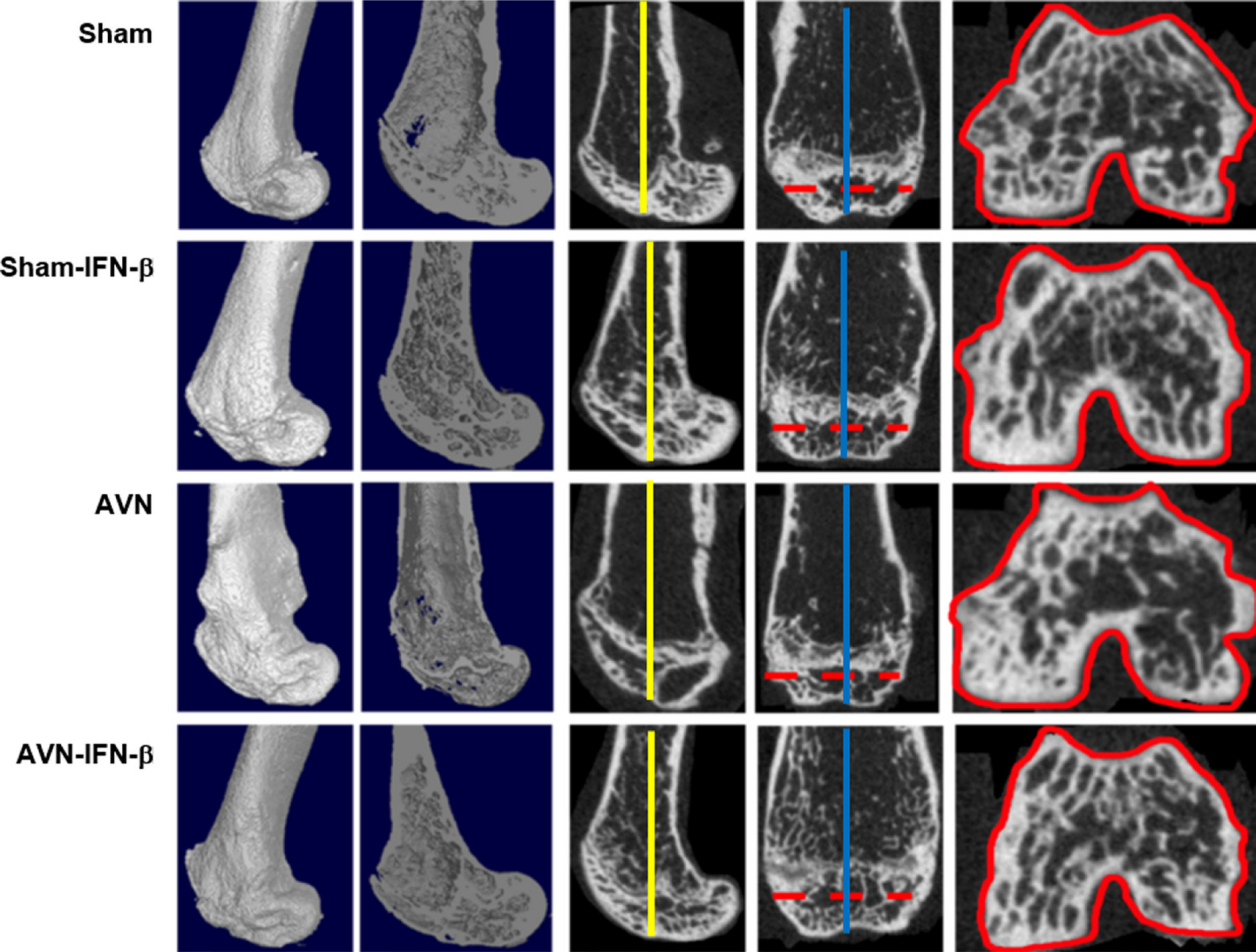
Representative micro-CT scan images of infarcted distal femurs in the sham-control, sham-IFN-β, AVN, and AVN- IFN-β groups Shown are distal femurs obtained from the infarcted area four weeks after surgery-induced avascular necrosis. The ROIs (solid red lines) of each group were obtained from the crosssectional area of the middle of the femoral epiphysis (dotted red lines). The sagittal images were formed by the cross-sectional area of the blue lines perpendicular to the dotted red lines, and the coronal images were formed by the cross-sectional area of yellow lines.

**Figure 2:**
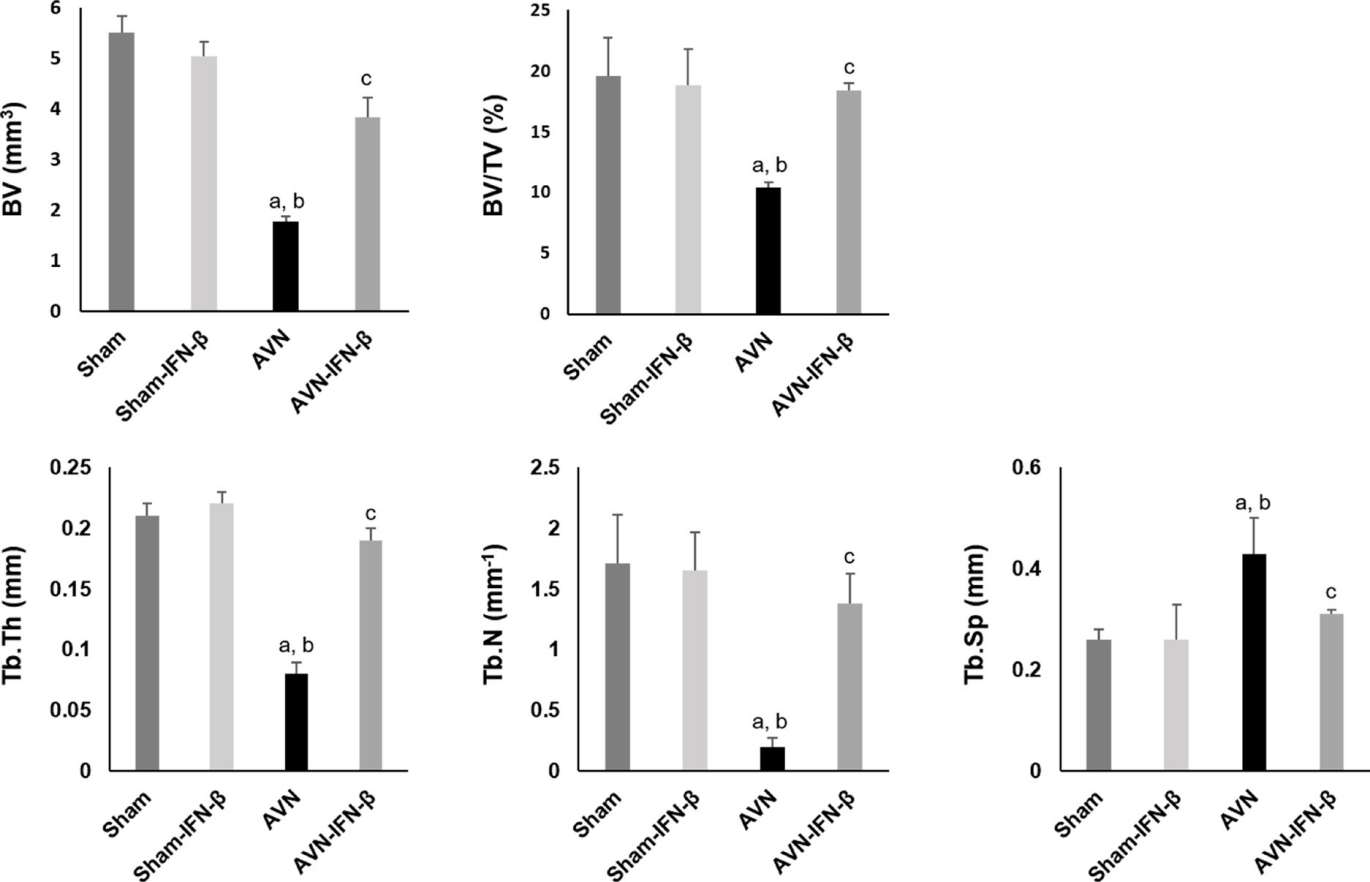
Effect of IFN-β on ischemic osteonecrosis of distal femur as assessed by micro-CT Values represent mean ± SEM for 5 animals in each group. BV, bone volume; TV, Tissue volume; Tb.Th, trabecular thickness; Tb.N, trabecular number; Tb.Sp, trabecular separation. ^**a**^*p <* 0.05 vs. Sham; ^**b**^*p <* 0.05 vs. Sham-IFN-b; ^**c**^*p <* 0.05 vs. AVN.

Micro-CT analysis was performed to evaluate a structural and quantitative assessment at the osteonecrotic site. Distal femoral epiphyses in the sham group animals did not develop osteonecrosis. Distal femoral epiphyses in the AVN group mice showed significant loss of bone and less preservation of architecture. In contrast, the trabecular bone and bone architecture were well preserved in the AVN-IFN-β animals (Figure 1). Micro-CT findings were quantified in Figure 2. The BV after surgery was significantly higher in the AVN-IFN-β group compared to the AVN group. The mean BV/TV values and trabecular numbers were significantly lower in the AVN group than in those of the AVN-IFN-β group, whereas the AVN-IFN-β group had bone masses and a microarchitecture that were similar to those of the sham group. Trabecular number and thickness were significantly higher and trabecular separation was significantly lower in the AVN-IFN-β group than in the AVN group. There were no significant differences between sham group and sham- IFN-β group.

### Osteoclastic resorption is attenuated by IFN-β

**Figure 3:**
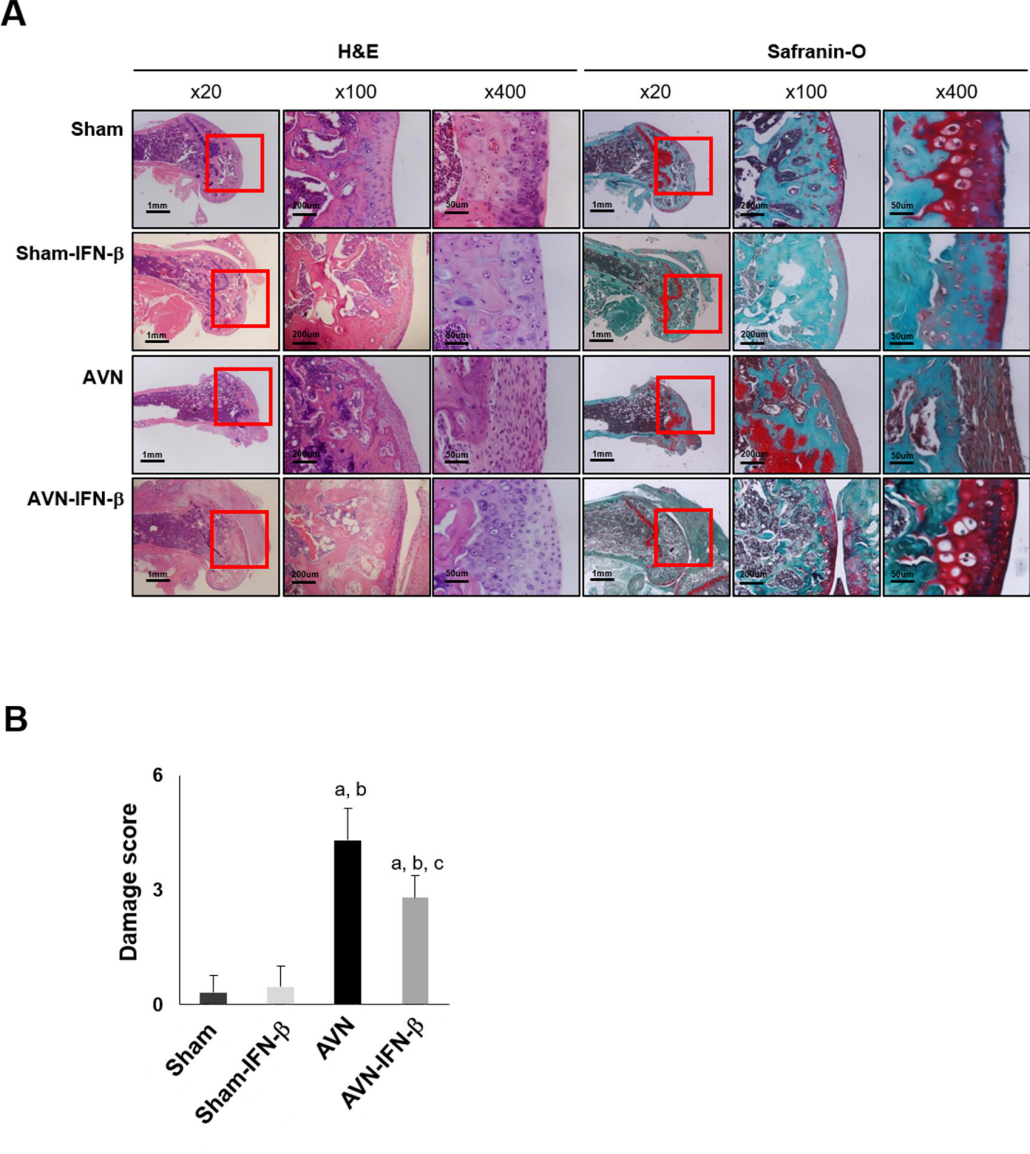
Histological findings in sham and AVN groups (**A**) Histology of distal femurs in representative H&E and Safranin-O stained sections are shown. Original magnification of the figures is indicated in the figure and magnified areas are indicated by red box (*n* = 25 mice for each group). (**B**) Damage scores are expressed as the mean ± SEM (*n =* 5 per group). ^**a**^*p <* 0.05 vs. Sham; ^**b**^*p <* 0.05 vs. Sham-IFN-β; ^**c**^*p <* 0.05 vs. AVN.

The distal femoral epiphyses of the sham and sham-IFN-β group animals were intact, had a normal joint surface and well-preserved distal femoral epiphyses. Whereas, distal femoral epiphyses in the AVN group showed obvious destruction compared with the sham- and sham-IFN-β group, and the epiphyseal joint cartilage secondary ossification centers were damaged and partly replaced by fibrous tissue. However, the distal femoral epiphyses of the AVN-IFN-β group mice were relatively saved compared to those in the AVN group (Figure 3A). Damage scores of the distal femoral epiphyses were significantly lower in the AVN-IFN-β group compared with the AVN group (*p* < 0.01) (Figure 3B).

**Figure 4:**
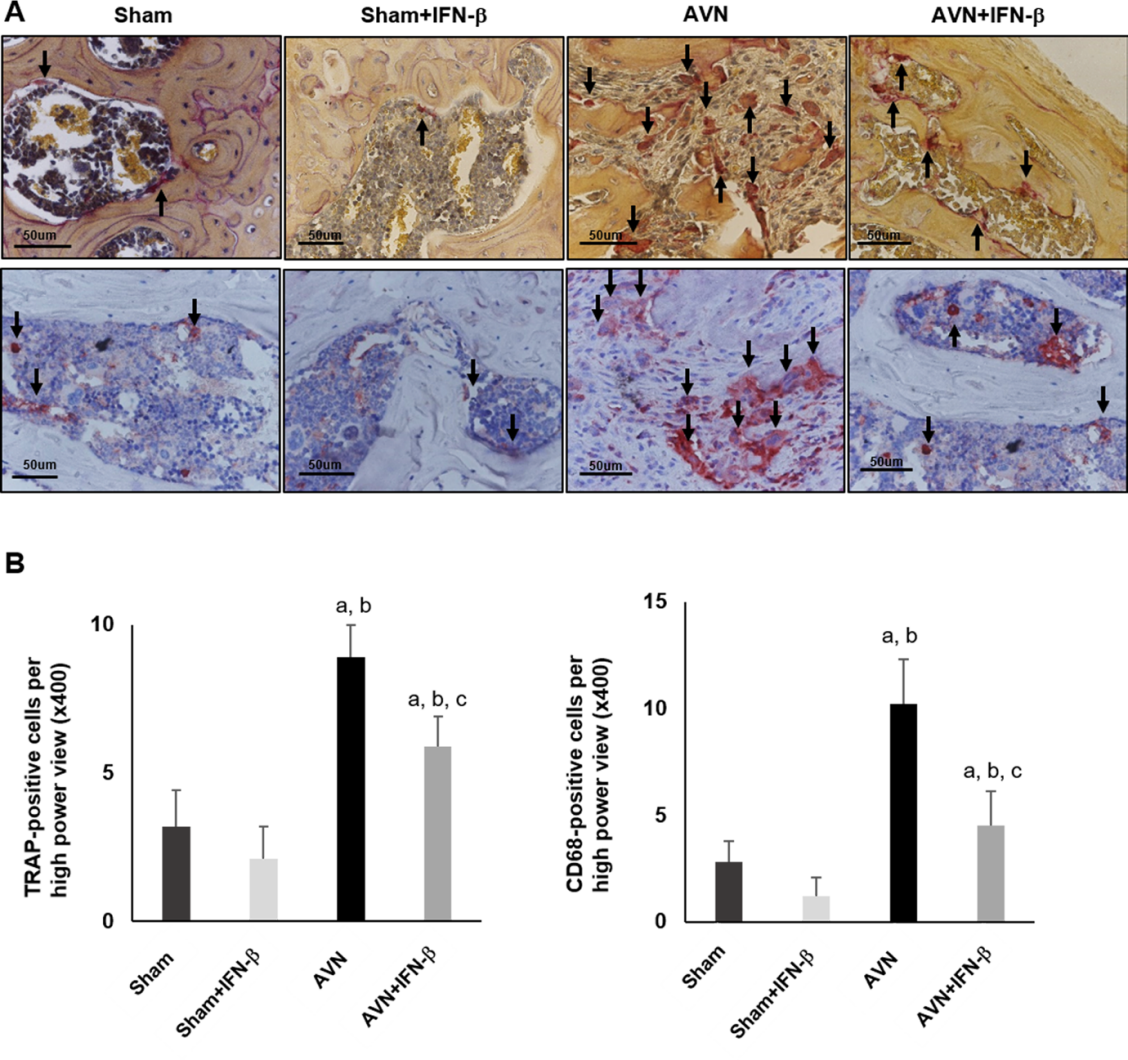
TRAP and CD68 staining in the distal femoral epiphysis (**A**) Representative sections from each group were stained with tartrateresistant acid phosphatase (TRAP) and CD68. Arrows indicate TRAP positive or CD68 positive osteoclasts. (**B)** TRAP-positive and CD68-positive osteoclast were counted from highest numbered high-power field (×400 magnification) in each section. Cell counts are expressed as mean ± SEM (*n =* 5 per group). Original magnification ×400. ^**a**^*p <* 0.05 vs. Sham; ^**b**^*p <* 0.05 vs. Sham-IFN-β; ^**c**^*p <* 0.05 vs. AVN.

When osteoclastic resorption were evaluated using TRAP staining and immunostaining for CD68, the number of TRAP- or CD68-positive cells was significantly higher in the AVN group than in the sham group and sham-IFN-β groups (Figure 4A). However, the osteoclastic resorption activity of the distal femoral epiphyses was decreased in the AVN-IFN-β group compared to that in the AVN group (Figure 4B).

**Figure 5:**
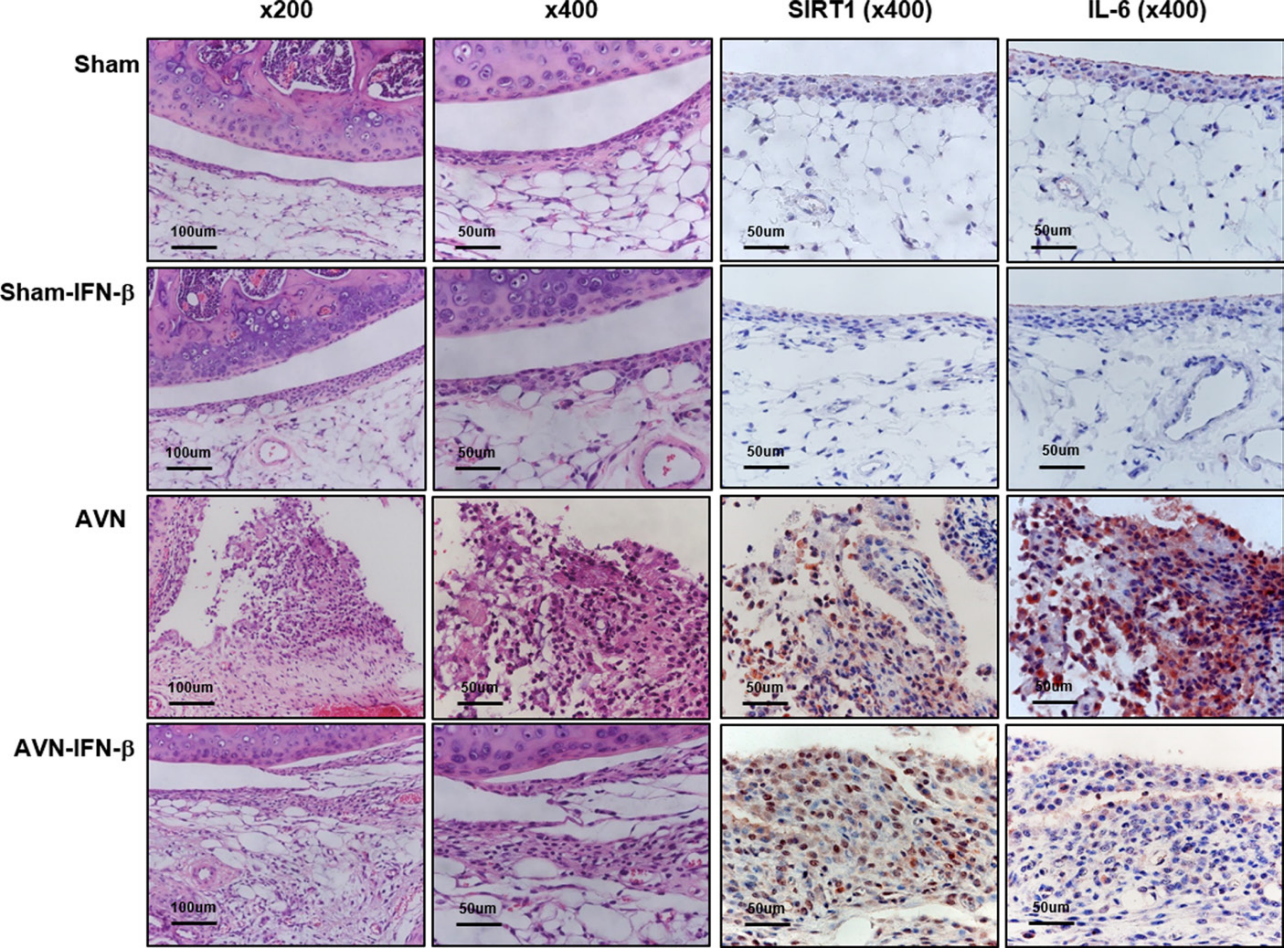
Histologic features of synovium Representative H&E stained section of synovial tissue from each group and immunohistochemical staining for SIRT1 and IL-6 was performed in each representative section.

To address the relationship between synovial inflammation and bone resorption, we performed histologic and immunohistologic evaluation for synovial tissue surrounding the epiphysis of the distal femur. The synovium in the AVN group showed synovial cell proliferation with prominent inflammatory cell infiltration compared to the sham and sham-IFN-β groups. However, the synovium of AVN-IFN-β group mice showed relatively less inflammatory cell infiltration compared to that in the AVN-control group (Figure 5).

### Treatment with IFN-β induces SIRT1 expression and inhibits IL-6 expression in an AVN animal model

**Figure 6:**
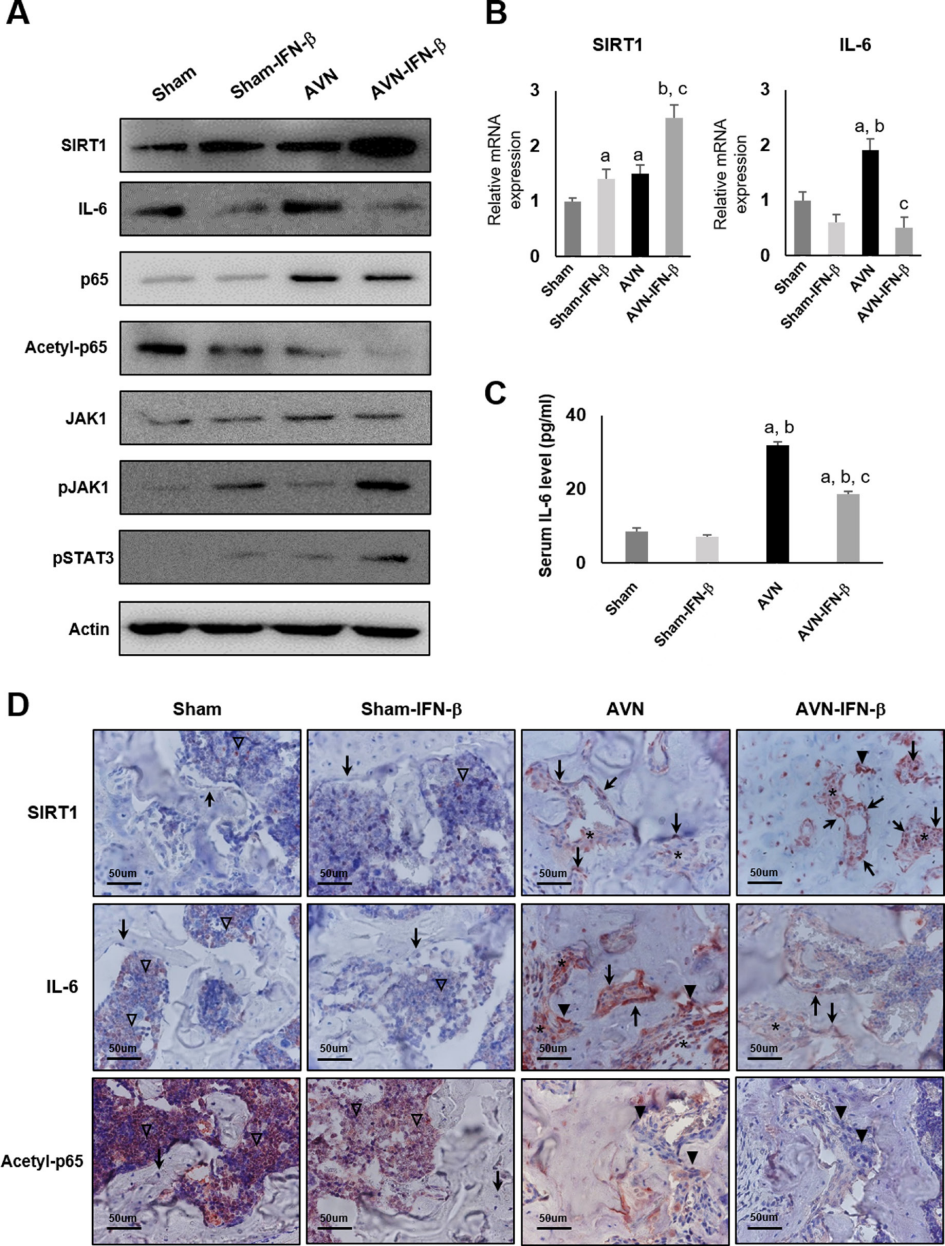
Western blot analysis, real-time RT-PCR, and immunohistochemical staining of sham and AVN groups four weeks after IFN-β treatment (**A**) Western blot analysis demonstrated SIRT1, IL-6, p65, acetylated p65, JAK1, pJAK1 and pSTAT3 expression in the distal femurs of the sham-control, sham-IFN-β, AVN, and AVN-IFN-β groups. (**B**) mRNA Expression of SIRT1 and IL-6 genes was analyzed via real-time RT-PCR. Data are expressed as the mean ± SEM (*n =* 5 per group). ^**a**^*p <* 0.05 vs. Sham; ^**b**^*p <* 0.05 vs. Sham-IFN-β; ^**c**^*p <* 0.05 vs. AVN. (**C**) Serum levels of the IL-6 level were measured by ELISA. (**D**) Immunohistochemical stained sections of SIRT1, IL-6 and acetyl-p65 in section from each group. Arrows indicate osteoblast, arrow heads indicate osteoclast, asterisks indicate stromal cells, and empty arrow head indicate hematopoietic cells in the marrow. Original magnification ×400.

We evaluated the expression of SIRT1 and IL-6 in an AVN animal model. In sham-treated animals, treatment with IFN-β induced SIRT1 expression but suppressed IL-6 expression. Additionally, the expression levels of SIRT1 and IL-6 were increased under ischemic conditions established in an AVN model (Figure 6A, 6B). Overall, following IFN-β treatment and AVN induction, the expression of SIRT1 mRNA and protein was highest in AVN-IFN-β group. However, the expression of IL-6 mRNA, protein, and serum level decreased following treatment with IFN-β, even in AVN-induced animals (Figure 6A–6C). In addition, the expression of acetylated p65 NF-kB decreased after IFN-β treatment in both the sham and AVN groups, despite no change in the expression of p65 (Figure 6A). The expression of acetylated p65 was lowest in the AVN-IFN-β group, which showed the highest level of SIRT1 expression. These findings might be the consequence of the deacetylase activity of SIRT1 for NF-kB. The expression of JAK1 was no change after IFN-β treatment. However, the expression of pJAK1 and pSTAT3 increased after treatment of IFN-β in both the sham and AVN groups. Therefore, we suggest that IFN-β treatment increases the expression of SIRT1 by activating the Janus kinase-signal transducer and activator of transcription (JAK-STAT) pathway.

Consistent with Western blot results, immunohistochemical expression of SIRT1 was higher in AVN-IFN-β groups and IL-6 expression was higher in the AVN group. The expression of acetylated p65 NF-kB decreased in the AVN-IFN-β groups. The expression of SIRT1 and IL-6 was seen in osteoclasts, osteoblasts, and stromal cells (Figure 6D). The expression of SIRT1 and IL-6 was very low in hematopoietic cells of marrow and undetectable in osteoblasts in the sham-control and sham-IFN-β groups.

### Suppression of IL-6 expression by IFN-β is mediated by SIRT1 in RAW264.7 cells, bone marrow derived osteoclasts, and MC3T3E1 cells

**Figure 7:**
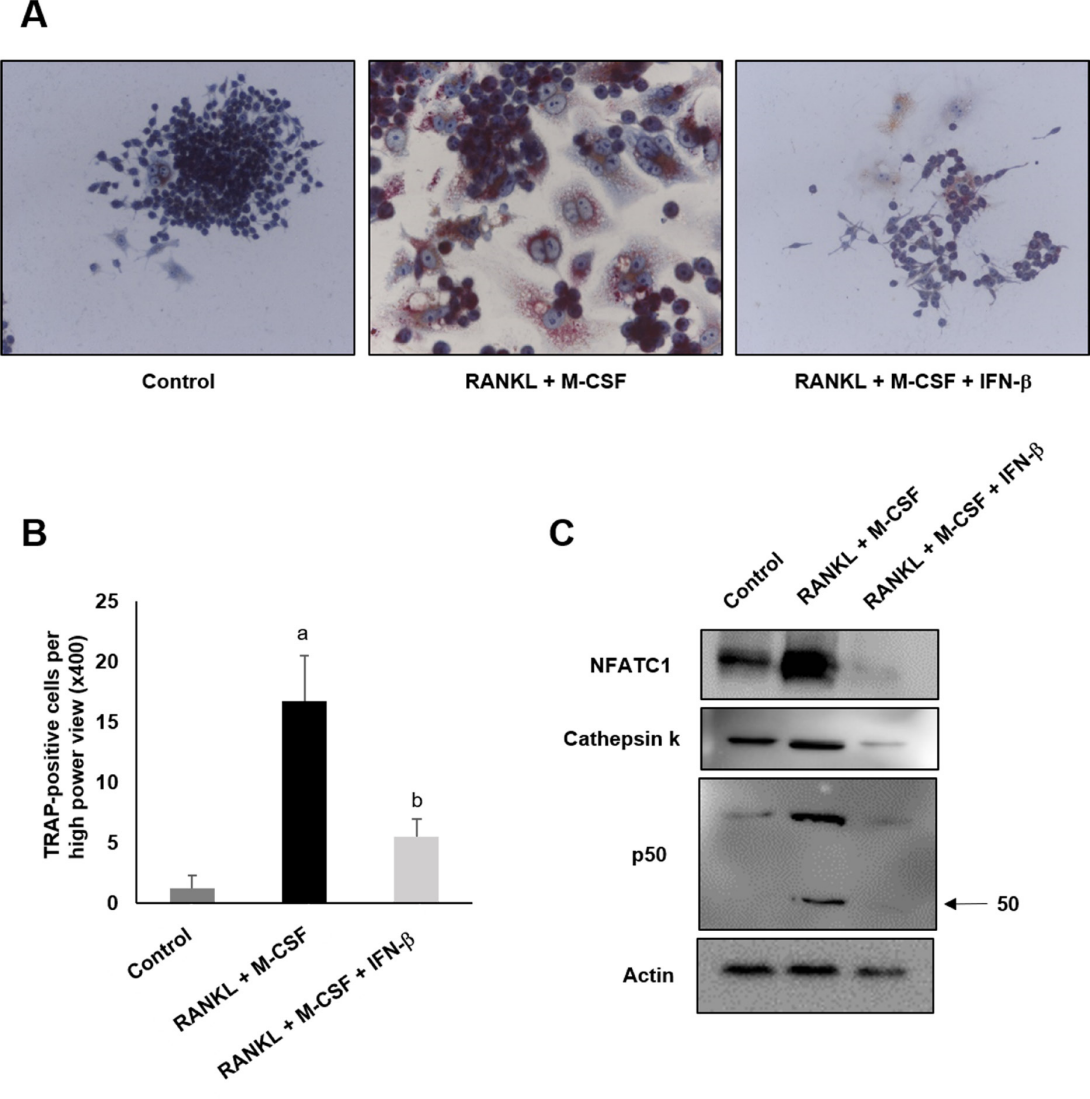
IFN-β inhibits osteoclast differentiation RAW 264.7 cells were stimulated to differentiate into osteoclast with 100 ng/ml of RANKL and 50 ng/ml of M-CSF with or without 200 U/ml of recombinant IFN-β and further cultured for 5 days. (**A**) RAW 264.7 cells were stained with tartrateresistant acid phosphatase (TRAP). (**B**) TRAP-positive cells were counted from highest numbered high-power field (×400 magnification) in each section. Cell counts are expressed as mean ± SEM. Original magnification ×400. ^**a**^*p <* 0.05 vs. control; ^**b**^*p <* 0.05 vs. RANKL + M-CSF. (**C**) After differentiation, the cells were harvested and Western blot analysis was performed to check the protein level of NFATC1, cathepsin K and p50.

IFN-β markedly suppressed osteoclast differentiation in RAW264.7 cells as assessed using TRAP staining. Five days after RANKL-induced osteoclast differentiation, IFN-β treatment decreased the number of TRAP-positive cells (Figure 7A, 7B) (*P* < 0.05). In addition, Western blot analysis showed IFN-β decreased the protein expression level of NFATC1, cathepsin K, and p50 that are known to be involved in osteoclast differentiation (Figure 7C).

**Figure 8:**
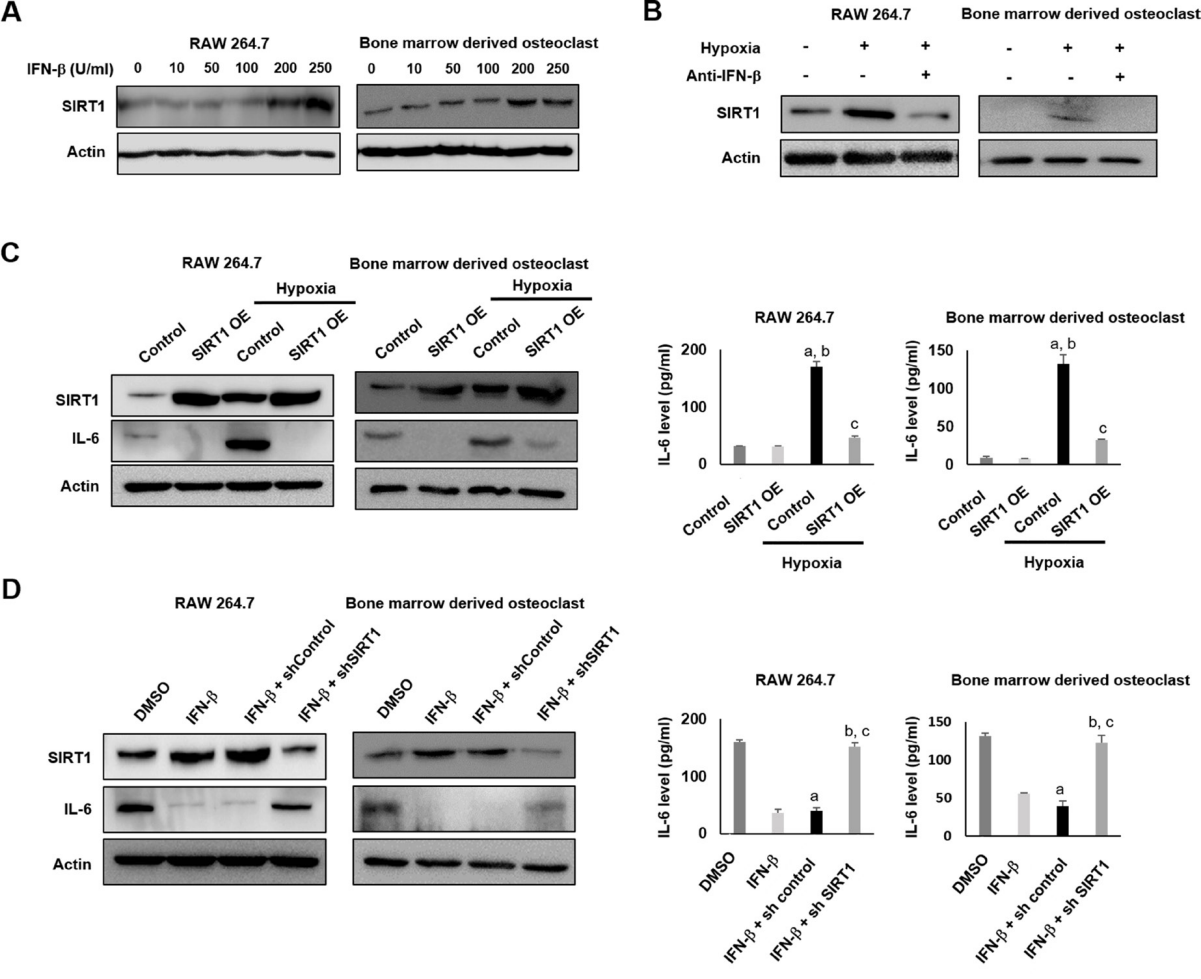
IFN-β inhibits hypoxia-induced expression of IL-6 in RAW 264.7 cells and bone marrow derived osteoclasts through SIRT1 upregulation (**A**) RAW 264.7 cells and bone marrow derived osteoclasts were incubated with the indicated doses of IFN-β for 12 h. IFN-β treatment induced SIRT1 expression at the dose at 200 U/ml. (**B**) The effect of IFN-β neutralizing antibody on hypoxia-induced SIRT1 expression. RAW 264.7 cells and bone marrow derived osteoclasts were incubated in hypoxic conditions in the presence or absence of IFN-β neutralizing antibody (1 mg/mL) for 24 h. (**C**) RAW 264.7 cells and bone marrow derived osteoclasts were transfected with either control or SIRT1 plasmid DNA in normoxic or hypoxic conditions. Western blotting was performed for SIRT1 and IL-6 using β-actin as a loading control (left panel). The level of IL-6 in cell culture supernatants was measured by ELISA (right panel). The ELISA results are expressed as the mean ± SEM (*n =* 5 per group). ^**a**^*p <* 0.05 vs. Sham; ^**b**^*p <* 0.05 vs. Sham-IFN-b; ^**c**^*p <* 0.05 vs. AVN. (**D**) RAW 264.7 cells and bone marrow derived osteoclasts were incubated under hypoxic conditions with treatment of IFN-β or DMSO. In addition, IFN-β-treated RAW 264.7 cells were transfected with either control or sh SIRT1. Western blotting was performed for SIRT1 and IL-6 using β-actin as a loading control (left panel). The level of IL-6 in cell culture supernatants was measured by ELISA (right panel). The ELISA results are expressed as the mean ± SEM (*n =* 5 per group). ^**a**^*p <* 0.05 vs. Sham; ^**b**^*p <* 0.05 vs. Sham-IFN-β; ^**c**^*p <* 0.05 vs. AVN.

As shown in Figure 3, IFN-β treatment affected the expression of both SIRT1 and IL-6. Therefore, we evaluated the molecular relationships between IFN-β, SIRT1, and IL-6 in hypoxic conditions. In RAW 264.7 cells and bone marrow derived osteoclasts, IFN-β dose dependently increased the expression of SIRT1 (Figure 8A) and hypoxia-induced expression of SIRT1 was attenuated by IFN-β-neutralizing antibody (Figure 8B). As the expression of SIRT1 and IL-6 increased in AVN model (Figure 6), the expression of both SIRT1 and IL-6 were increased in hypoxic control cells compared with normoxic condition (Figure 8C). However, overexpression of SIRT1 inhibited IL-6 mRNA and protein expression in both normoxic and hypoxic conditions (Figure 8C). These findings suggest that the expression of SIRT1 might be regulated by IFN-β in hypoxia and that the expression of IL-6 suppressed by SIRT1. However, the direct relationship between the expression of IFN-β and IL-6 was not clear. Therefore, we evaluated the effect of IFN-β on the regulation of IL-6 expression under conditions of SIRT1 suppression. As shown in Figure 8D, the suppressive effect of IFN-β on IL-6 expression was attenuated by knock-down of SIRT1 using shRNA for SIRT1 in RAW 264.7 cells and bone marrow derived osteoclasts. There were no significant changes in the hypoxia-induced release of IL-6 in RAW 264.7 cells and bone marrow derived osteoclasts after the knockdown of SIRT1 and IFN-β treatment. These data suggest that IFN-β contributes to the negative modulation of hypoxia-mediated inflammatory signaling in RAW 264.7 cells and bone marrow derived osteoclasts through SIRT1 upregulation.

On the other hand, in osteoblastic MC3T3E1 cells, IFN-β treatment increased the expression of SIRT1 under normoxic condition. However, there was no significant effect of IFN-β on SIRT1 expression in hypoxic condition and IL-6 expression in both normoxic and hypoxic conditions. Moreover, we analyzed the level of IL-6 in cell culture supernatants of MC3T3E1 cells with ELISA. IFN-β had limited effect on IL-6 level both in normoxic and hypoxic conditions. (Supplementary Figure 1).

## DISCUSSION

**Figure 9:**
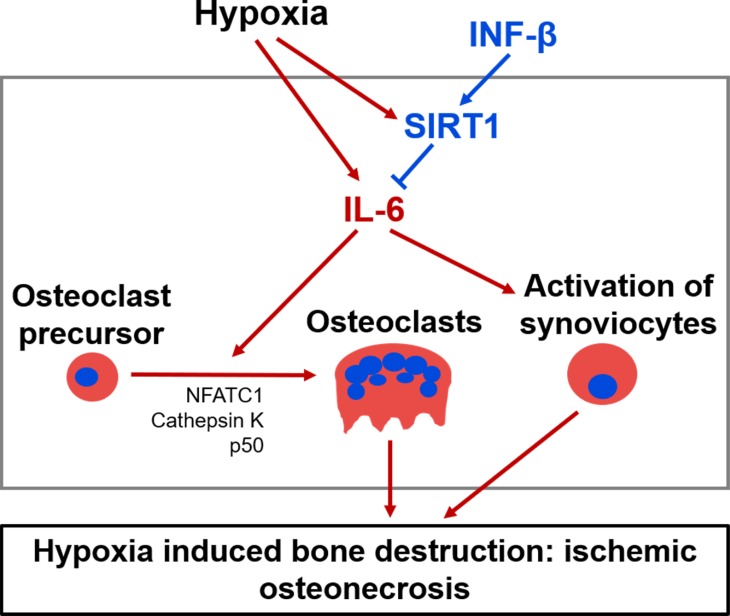
A proposed mechanism for IFN-β protection against AVN The administration of exogenous IFN-β suppresses the secretion of the proinflammatory cytokine IL-6 mediated by stimulating SIRT1 expression. IFN-β reduces inflammation and protects against bone destruction.

In this study, we demonstrated that IFN-β protects against AVN in a mice model by suppressing the secretion of the proinflammatory cytokine IL-6. Thus, the present study demonstrates for the first time that exogenous IFN-β, starting at the onset of disease, in the murine AVN model reduces inflammation and protects against bone destruction. Moreover, we have shown that the effect of IFN-β on the suppression of IL-6 is mediated by its role in stimulating SIRT1 expression (Figure 9). Therefore, in addition to the protective role of IFN-β itself for the AVN, our results suggest that SIRT1 or IL-6 also might be used as therapeutic targets of AVN for the development of pharmacological drug to protect against AVN.

IL-6 is a pathognomonic inflammatory cytokine present in the synovium at the active stage of AVN [18]. Yamaguchi R. *et al.* reported that the production of IL-6 from articular chondrocytes was due to HIF-1α activation, which stimulates an inflammatory response from synovial cells. Moreover, they suggested the therapeutic potential of an IL-6 blockade for decreasing synovitis following ischemic osteonecrosis [18]. In inflammatory disorders such as rheumatoid arthritis, IL-6 has been shown to enhance the formation and activity of osteoclasts [19, 20] and inhibit the formation and activity of osteoblasts [20, 21]. Therefore, based on our results and previous studies, we suggest that IL-6 is an important factor for osteoclastic resorption in early stage of ischemic osteonecrosis.

A recent study demonstrated that IFN-β increased SIRT1 expression by activating the Janus kinase-signal transducer and activator of transcription (JAK-STAT) pathway in mouse bone marrow derived macrophages [22]. In addition, anti-inflammatory effect of IFN-β through SIRT1 upregulation and down regulation of inflammatory cytokines including IL-6 has been investigated in lethal endotoxic and septic shock [22]. Furthermore, systemic treatment of IFN-β resulted in inhibition of cartilage and bone erosion cause by a significant decrease in TNF and IL-6 expression [17]. While it is well established that SIRT1 is involved in inflammatory process, its role in AVN has never been studied. Therefore, we have an interest in the IFN-β/SIRT1/IL-6 axis for treating AVN. In this study, we found that IFN-β inhibited the AVN-induced secretion of the proinflammatory cytokine IL-6 via SIRT1 upregulation. Consistent with previous study, our results showed that IFN-β increases SIRT1 expression by activating the JAK-STAT pathway. We suspect that the inhibitory effect of IFN-β on IL-6 preserved bone architecture due to decreased osteoclastic bone resorption. However, there was no significant effect of IFN-β on SIRT1 expression in osteoblasts and IL-6 expression in osteoblasts in both normoxic and hypoxic conditions. Therefore, we suggest that the effect of IFN-β on the suppression of IL-6 mediated by stimulating SIRT1 expression occurs mainly in osteoclasts.

Various studies using animal models of osteonecrosis support the hypothesis that inhibition of bone resorption after osteonecrosis can preserve the trabecular framework of the necrotic bone and minimize deformity [23]. Previous studies have shown that bisphosphonate, an antiresorptive agent, retarded bone destruction in adult osteonecrosis [24]. However, a recent study demonstrated that use of zoledronate for AVN with severe involvement of necrotic area did not prevent the collapse of the femoral head or reduce the need for total hip arthroplasty [25]. In addition to the bisphosphonate studies, RANKL inhibition has also been shown to decrease bone resorption after ischemic osteonecrosis [23]. However, one concern about using antiresorptive agent therapies to treat AVN is that preservation of the necrotic bone without bone turnover and new bone formation may lead to necrotic bone collapse over time [2]. Although our results showed well preserved necrotic bone after IFN-β treatment, the effect of IFN-β in AVN is mainly to inhibit osteoclastic resorption without stimulation of new bone formation. Therefore, we suggest that combined therapy with reduction of bone resorption and stimulation of bone formation to improve bone volume and the mechanical strength of necrotic bone might be clinically useful.

Ideal injection time to AVN is as early as possible before bony collapse occurs. However, a recent study reported that there were necrotic blood vessels without neovascularization until 2 weeks following AVN induction in the distal femoral epiphysis in a mouse model [26]. During this period, there is no blood supply to the distal femoral epiphysis. Kamiya *et al.* demonstrated that vascular tissue invasion of the necrotic marrow space was observed at 2 weeks after AVN induction [25]. Hence, we thought that IFN-β might not reach the distal femoral epiphysis during early IFN-β administration. Therefore, we injected IFN-β at 2 weeks post AVN induction after regeneration of the blood supply to the necrotic area. Inflammation not only affects osteoclast activity but also angiogenesis, and revascularization which are critical for bone regeneration in affected areas. According to a recent report, revascularization of necrotic epiphysis begins 2 weeks after AVN induction [26]. Although we did not investigate revascularization, we suggest that IFN-β be systemically delivered into necrotic areas through regenerated blood vessels.

A limitation of this study was that we only investigated the relationship between SIRT1 and IL-6. Further detailed investigation of the mechanism of the therapeutic role of IFN-β in synovitis and bone remodeling following AVN is warranted. Another limitation was that we analyzed the deformity caused by epiphysis collapse epiphysis 4 weeks after AVN induction. According to a previous report, epiphysis collapse occurred 6 weeks after AVN induction. Therefore, it would be better to perform analysis of epiphyseal deformity 6 weeks after AVN induction. Another limitation was that we used the distal femoral AVN model while AVN mainly occurs in the proximal femur; nevertheless, our mouse model of AVN in the distal femoral epiphysis was recently developed as a model investigating the mechanisms of AVN.

Currently, there are no specific treatments for AVN other than surgical treatment such as arthroplasty. IFN-β may have positive effects in terms of improving the remodeling of the necrotic bone. Overall, the present study is the first report demonstrating the mechanism of the protective effect of IFN-β on AVN via SIRT1 upregulation. Our results suggest that IFN-β and its downstream target, SIRT1, have important roles in anti-inflammatory responses and may be potential targets for treatment in the early stage of disease to prevent osteoclastic bone resorption.

## MATERIALS AND METHODS

### Animals and surgical procedures

Seven- to eight-week old C57bl/6 male mice were used in the study. Mice had free access to water and standard mouse chow pellets and were housed under controlled temperature (22 ± 1°C) and humidity (50 to 60%) with a 12-hour light-dark cycle from 7 AM to 7 PM. AVN was surgically induced in the distal femur of mice using a dissection microscope and the previously described technique [26]. Beginning on Day 14 after the induction of AVN, the intervention group mice received 10,000 IU of exogenous mouse IFN-β (PBL Assay Science, Piscataway, NJ, USA) every day by intraperitoneal injection for 4 days, while control group mice were similarly treated with sterile saline. The animals were divided into the following groups: 1) the sham-operated group (sham group), 2) the sham-operated, IFN- β-injected group (sham-IFN-β group), 3) the AVN group (AVN group), and 4) the AVN, IFN-β-injected group (AVN-IFN-β group) (*n =* 25/group).

Animals were euthanized by exsanguination under sodium pentobarbital anesthesia 4 weeks after inducing AVN. Animals were cared for in accordance with the National Institutes of Health Guidelines for Animal Care. All experimental procedures were approved by the Institutional Animal Care and Use Committee at Chonbuk National University (approval number: CBNU 2016–28).

### Assessment of bone destruction by MicroCT

A SKYSCAN 1076 Micro-CT unit (Skyscan, Kontich, Belgium) was used to assess bone volume within defect sites. The X-ray source was set at 75 kV and 100 mA, with a pixel size of 8.8 mm and 400 projections were acquired over an angular range of 180 u (angular step of 0.45 u). The area included in CT scans was distal half of the femur from the middle margin of the femoral diaphysis to the distal femoral epiphysis. A global thresholding algorithm was applied at a constant threshold for all specimens. The threshold was the intensity (gray value) that corresponded to, 45% of the average intensity of the intact cortical bone in specimens. Voxels with intensities exceeding the threshold were considered to contain mineralized tissue. All system aspects were operated using Dataviewer software (SkyScan). On stacked, reconstructed micro-CT cross-section images, manual regions of interest (ROIs) of irregular anatomical contour were drawn on transverse images at the middle of the femoral condyle (Figure 1). ROIs excluded cortical bone. The volume of interest (VOI) was a stack of ROIs drawn over 52 cross-sections, resulting in a height of 0.45 mm. Tb.Th was the trabeculae mean thickness, Tb.Sp was the mean distance between trabeculae and Tb.N was the average number of trabeculae present per unit length. Tb.Th and Tb.Sp were assessed using direct 3D methods and Tb.N was calculated using the formula Tb.N = (BV/TV)/Tb.Th.

### Histological methods and evaluation of bone resorption activity

Resected femurs were fixed in 10% neutral buffered formalin and decalcified in 10% EDTA for 10 days or in rapid decalcifying solution (Calci-Clear Rapid) (National Diagnostics, Atlanta, GA, USA) for 12 h. Tissue sections were from the femoral midline as the most representative area. To evaluate histologic findings, paraffin-embedded tissue sections were stained with hematoxylin and eosin or Safranin-O staining (Sigma-Aldrich, St Louis, MO, USA). Femoral damage was graded from 0 to 5 as: 0, no damage; 1, mild damage with maintaining femoral head articular cartilage and architecture; 2, destruction of the articular cartilage and collapse of the secondary ossification center but with less than one-third of the femoral head showing destruction; 3; destruction of between one-third and two-thirds of the femoral head; 4, destruction of more than two-thirds of the femoral head; and 5, complete or near-complete femoral head destruction [2, 3].

### Tartrate-resistant acid phosphatase (TRAP) staining

The paraffin-embedded sections of the bones of the mice and RANKL-induced osteoclastogenesis on the fourth weeks after induction were gently washed twice with pre-warmed, double-distilled water (37°C), fixed with stationary liquid for 20 sec, and stained with tartrateresistant acid phosphatase (TRAP) (Sigma-Aldrich, St Louis, MO, USA) for 60 min at 37°C. The TRAP-stained cells were then gently washed, counterstained in the dark with hematoxylin or 100 μL/well of 300 nM diamidino-2-phenylindole (DAPI) in phosphate buffer solution (PBS) containing 0.1% Triton X-100 at room temperature for 15 min, and examined with a ZEISS Vert.A1 microscope (Carl Zeiss, Oberkochen, Germany). TRAP-positive cells appeared dark red, and TRAP-positive multinucleated cells containing three or more nuclei were counted as osteoclasts. [16]. To investigate osteoclast bone resorption activity in the distal femur, TRAP-positive multinucleated cells containing three or more nuclei were counted at highest numbered five high-power fields ×400 magnification) in each femur. All experiments were carried out in triplicate at least 3 times.

### Immunohistochemical staining

After deparaffinizing, sections were treated with sodium citrate buffer in a pressure cooker for 12 min to facilitate antigen retrieval. After blocking endogenous peroxidase, sections were incubated with Protein Block Serum-Free (DAKO, Carpentaria, CA, USA) at room temperature for 10 min to prevent nonspecific staining and then incubated with antibody against SIRT1 (1:100 dilutions, Santa Cruz Biotechnology, Santa Cruz, CA, USA), or IL-6 (1:500 dilutions, Novus Biologicals, Littleton CO, USA), CD68 (DAKO, Carpentaria, CA, USA) or acetyl-p65 (1:100 dilutions, Cell Signaling Technology, Beverly, MA, USA) overnight at 4°C. Peroxidase activity was detected using the enzyme substrate 3-amino-9-ethylcarbazole.

### Western blot analysis

The distal femoral epiphysis was harvested and decalcified in rapid decalcifying solution (Calci-Clear Rapid) for 12 h. Soft tissue were trimmed from the specimens, washed with phosphate buffered saline to removed contaminants, and then ground to a fine powder using a manual biopulverizer cooled in liquid nitrogen. Total cell extracts were generated by harvesting in a lysis buffer (Cell Signaling, Beverly, MA, USA) and then centrifugation at 12,000 × g for 15 min at 4°C. Quantification of total protein was performed with BCA protein assay reagent (Bio-Rad Laboratories, Hercules, CA, USA). Proteins were resolved on a 10% SDS-PAGE gel and transferred to a PVDF membrane. After blocking in 5% milk in Tris-buffered saline with 0.1% Tween-20 (TBST), the membrane was incubated with primary antibodies specific for SIRT1 (Santa Cruz Biotechnology, Santa Cruz, CA, USA), IL-6 (Novus Biologicals, Littleton CO, USA), p65 (Abcam, Cambridge, MA, USA), NFATC1 (Santa Cruz Biotechnology, Santa Cruz, CA, USA), Cathepsin K (Abcam, Cambridge, MA, USA), p50 (Cell Signaling Technology, Beverly, MA, USA), acetyl-p65 (Cell Signaling Technology, Beverly, MA, USA), JAK1 (Santa Cruz Biotechnology, Santa Cruz, CA, USA), pJAK1 (Cell Signaling Technology, Beverly, MA, USA), pSTAT3 (Cell Signaling Technology, Beverly, MA, USA) and β-Actin (Santa Cruz Biotechnology, Santa Cruz, CA, USA). After washing, the blots were incubated with secondary antibody diluted 1:5,000 in TBS-T at room temperature for 1 h. Signals were detected by an enhanced chemiluminescence (ECL) reagent (Santa Cruz Biotechnology, Santa Cruz, CA, USA), according to the manufacturer’s instructions. Densitometric analysis was performed directly from the blotted membrane using the LAS-3000 luminoimage analyzer system (Fujifilm, Tokyo, Japan). The relative phosphorylation level of each protein was calculated as the ratio of phosphorylated to total protein levels. At least five samples from each group were used.

### RNA preparation

Five mice from each group were evaluated. Total RNA was prepared from the distal femoral epiphysis using TRIzol reagent (Invitrogen, Carlsbad, CA, USA). Tissue harvesting was performed with a distractor for precision and accuracy. All extraneous soft tissue was cleaned and tissues were snap frozen in liquid nitrogen.

### Real time RT-PCR

RNA from extracted tissues was precipitated with isopropanol and dissolved in DEPC-treated distilled water. Total RNA (500 ng) was treated with RNase-free DNase (Invitrogen) and first-strand cDNA was generated using random hexamer primers supplied in first-strand cDNAsynthesis kits according the manufacturer’s protocol (Applied Biosystems, Foster City, CA, USA). Specific primers were designed (Table 1) using Primer Express software (Applied Biosystems). GAPDH sequence was used as an invariant control. Real-time RT-PCR reaction mixtures were 10 ng reverse-transcribed total RNA, 2 nM forward and reverse primers, and 5 3 PCR master mixture in 10 mL. Reactions were performed in 384-well plates using the ABI Prism 7900 HT Sequence Detection System (Applied Biosystems, Foster City, CA, USA). Samples from 5 mice were analyzed from each group and RNA from individual rats was analyzed separately.

**Table 1:** Sequences and accession numbers for forward (FOR) and reverse (REV) primers used in real-time RT-PCR

Gene		Primer sequence	Product size	Accession No.
Sirtuin 1	forward	AGTTCCAGCCGTCTCTGTGT	198	NM_019812.3
	reverse	CTCCACGAACAGCTTCACAA		
Interleukin 6	forward	AGTTGCCTTCTTGGGACTGA	159	NM_031168.2
	reverse	TCCACGATTTCCCAGAGAAC		
GAPDH	forward	AACTTTGGCATTGTGGAAGG	223	NM_001289726.1
	reverse	ACACATTGGGGGTAGGAACA		

### Cell culture and IFN-β treatment

The murine macrophage cell line RAW 264.7 cells (purchased from the Korean Cell Line Bank, Seoul, Korea) and MC3T3E1 cells (purchased from the Korean Cell Line Bank, Seoul, Korea) was maintained in Dulbecco’s modified Eagle’s medium (DMEM, Gibco BRL, Gaithersburg, MD, USA) and alpha-modified minimum essential medium, respectively (alpha-MEM, Gibco BRL, Gaithersburg, MD, USA) supplemented with 10% fetal bovine serum (Gibco BRL, Gaithersburg, MD, USA), and 1% Gibco™ Antibiotic-Antimycotic (100 U/ml penicillin, 100 microgram/ml of streptomycin, and 0.25 ug/ml of Fungizone (Gibco BRL, Gaithersburg, MD, USA) at 37°C in a humidified incubator with 5% CO_2_. To grow RAW 264.7 cells in hypoxic condition, cells were grown in cell culture plates inside an Oxoid anaerobic jar with an AnaeroGen™ sachet (ThermoFisher Scientific, Waltham, MA, USA). The AneroGen sachet in a sealed jar rapidly absorbs atmospheric oxygen with the production of carbon-dioxide and the oxygen concentration falls to below 1% within 30 minutes.

A dose-dependent experiment was conducted by treating RAW 264.7 cells with different concentration of INF-β. Western blot results revealed that 200 U/ml of INF-β activated SIRT1 (Figure 3A). We used this concentration to stimulate SIRT1 expression in subsequent experiments.

### Primary cell culture

Bone marrow cells were harvested from tibia and femur of 8- to 10-week-old C57bl/6 male mice. To generate osteoclast, these cells were cultured in alpha-modified minimum essential medium (Gibco BRL, Gaithersburg, MD, USA) containing 10% fetal bovine serum (Gibco BRL, Gaithersburg, MD, USA), and 1% Gibco™ Antibiotic-Antimycotic (100 U/ml penicillin, 100 microgram/ml of streptomycin, and 0.25 ug/ml of Fungizone, Gibco BRL, Gaithersburg, MD, USA) in the presence of RANKL (30 ng/ml, Sigma-Aldrich, St Louis, MO, USA) and of M-CSF (15 ng/ml, Sigma-Aldrich, St Louis, MO, USA) at 37°C in a humidified incubator with 5% CO_2_.

### *In-vitro* differentiation

RAW 264.7 cells were seeded in 48-well plate (5,000 cells/well) containing DMEM supplemented with 10% fetal bovine serum (Gibco BRL, Gaithersburg, MD, USA), and 1% Gibco™ Antibiotic-Antimycotic (100U/ml penicillin, 100 microgram/ml of streptomycin, and 0.25 ug/ml of Fungizone, Gibco BRL, Gaithersburg, MD, USA). Cells were stimulated to differentiate into osteoclast with 100 ng/ml of RANKL (Sigma-Aldrich, St Louis, MO, USA) and 50 ng/ml of M-CSF (Sigma-Aldrich, St Louis, MO, USA) with or without recombinant 200U/ml of IFN- β (PBL Assay Science, Piscataway, NJ, USA) and further cultured for 5days. The culture medium was replaced every day.

### Measuement of IL-6 in plasma and culture medium

Whole blood was collected from each mouse at 5 days after AVN surgery through tail vein puncture. Blood was collected into BD Vacutainer spray-coated K2 EDTA tubes (BD Diagnostic Systems, Franklin Lakes, NJ, USA), and plasma was isolated from anticoagulated blood after centrifugation. Also, culture medium from bone marrow derived osteoclasts were harvested. Commercial Enzyme-linked immunosorbent assay (ELISA) kits were used to measure mouse IL-6 (ELISA Ready-SET-Go, eBioscience, San Diego, CA, USA) according to the manufacturer’s instructions.

### SIRT1 activation and inhibition experiments.

SIRT1 plasmid DNA (pDNA) (Addgene, plasmid 1769, Cambridge, MA) was cloned into pCMV-tag2B (Stratagene, LaJolla, CA, USA). The same plasmid without the SIRT1 DNA insert was used as a control vector (CV). The plasmid used to express SIRT1 short hairpin RNA (shRNA) and CV were kindly provided by A. Brunet (Stanford University, Stanford, CA, USA). All plasmids were amplified in DH5a´ *Escherichia coli* competent cells (Invitrogen, Carlsbad, CA, USA) and were purified using an endo-free plasmid mega-prep kit (Qiagen, Valencia, CA, USA). Transient transfection of RAW 264.7 cells with SIRT1 pDNA and shRNA was performed using Lipofectamin 2000 (Invitrogen, Carlsbad, CA, USA). Forty-eight hours after transfection, the cells were harvested and used for further experiments.

### Statistical analysis

Statistical analysis was performed using one-way ANOVA followed by a posthoc test. Data are expressed as mean ± SEM. Differences with *p* values < 0.05 were considered statistically significant.

## SUPPLEMENTARY MATERIALS FIGURE


