# Supplementary material for: Interferon β protects against avascular osteonecrosis through interleukin 6 inhibition and silent information regulator transcript-1 upregulation
Source: Oncotarget. 2017 Dec 16;9(3):3562–75. doi: 10.18632/oncotarget.23337 (PMC5790483; doi:10.18632/oncotarget.23337)
Supplement: Supplementary file 1 [file oncotarget-09-3562-s001.pdf]

Interferon β protects against avascular osteonecrosis through interleukin 6 inhibition and silent information regulator transcript-1 upregulation

SUPPLEMENTARY MATERIALS

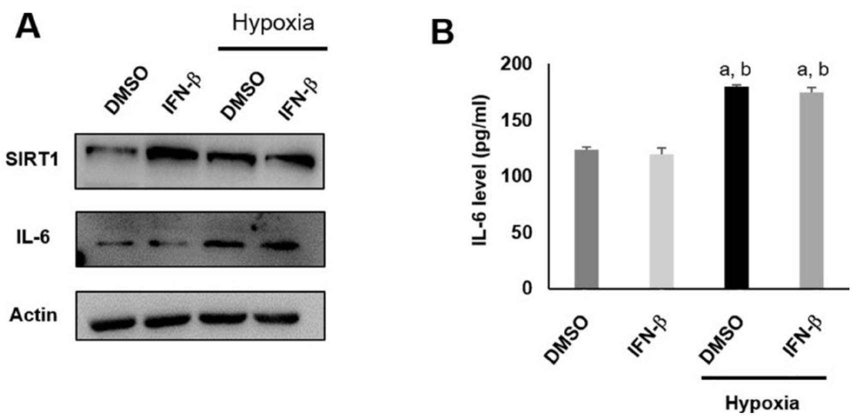

Supplementary Figure 1: IFN-β showed limited effects on the expression of SIRT1 and IL-6 in the MC3T3E1 osteoblast cell line. MC3T3E1 cells were incubated under normoxic or hypoxic conditions and treated with IFN-β or DMSO. (**A**) Protein levels of SIRT1 and IL-6 were analyzed by Western blotting. (**B**) Supernatants of MC3T3E1 cells were collected and IL-6 level was measured by ELISA.
